# Effects of Printing Layer Orientation on the High-Frequency Bending-Fatigue Life and Tensile Strength of Additively Manufactured 17-4 PH Stainless Steel

**DOI:** 10.3390/ma16020469

**Published:** 2023-01-04

**Authors:** Hamed Ghadimi, Arash P. Jirandehi, Saber Nemati, Huan Ding, Abdelrahman Garbie, Jonathan Raush, Congyuan Zeng, Shengmin Guo

**Affiliations:** 1 Department of Mechanical and Industrial Engineering, Louisiana State University, Baton Rouge, LA 70803, USA; 2 Department of Mechanical Engineering, University of Louisiana at Lafayette, Lafayette, LA 70503, USA; 3 Department of Mechanical Engineering, Southern University, Baton Rouge, LA 70807, USA

**Keywords:** additive manufacturing, atomic diffusion additive manufacturing (ADAM), bound powder extrusion (BPE), printing layer orientation, high-cycle fatigue, high-frequency bending fatigue, tensile strength

## Abstract

In this paper, small blocks of 17-4 PH stainless steel were manufactured via extrusion-based bound powder extrusion (BPE)/atomic diffusion additive manufacturing (ADAM) technology in two different orientations. Ultrasonic bending-fatigue and uniaxial tensile tests were carried out on the test specimens prepared from the AM blocks. Specifically, a recently-introduced small-size specimen design is employed to carry out time-efficient fatigue tests. Based on the results of the testing, the stress–life (S-N) curves were created in the very high-cycle fatigue (VHCF) regime. The effects of the printing orientation on the fatigue life and tensile strength were discussed, supported by fractography taken from the specimens’ fracture surfaces. The findings of the tensile test and the fatigue test revealed that vertically-oriented test specimens had lower ductility and a shorter fatigue life than their horizontally-oriented counterparts. The resulting S-N curves were also compared against existing data in the open literature. It is concluded that the large-sized pores (which originated from the extrusion process) along the track boundaries strongly affect the fatigue life and elongation of the AM parts.

## 1. Introduction

As new technologies are developed, and existing ones are improved, additive manufacturing (AM) [1,2] applications are expanding. Various AM technologies allow users to select the best one for their needs and applications. These AM techniques can be classed according to several factors, such as the material used (i.e., metal, polymer), feedstock type (i.e., powder, rod, filament), the energy source used (i.e., laser, E-beam, arc, friction), size of the parts or deposition rate (small/large-scale), printing environment (i.e., open-air, gas shield, inert gas), etc. For metal AM, common methods include laser fusion techniques (i.e., laser power bed fusion, L-PBF, [3,4,5,6], selective laser sintering/melting, SLS/SLM, and direct metal laser sintering, DMLS), bound metal filament extrusion-based fabrications [7] (i.e., bound powder extrusion/deposition, BPE, or atomic diffusion additive manufacturing, ADAM), as well as fused deposition modeling (FDM) [8,9,10]), friction-based AM (i.e., additive friction stir deposition, AFSD [11]), powder adhesion AM (i.e., metal injection molding, MIM), etc.

Bound powder extrusion/deposition (BPE) or atomic diffusion additive manufacturing (ADAM) [12,13] is an open-air extrusion-based metal AM technology that uses bound metal powder filament to print AM parts layer-by-layer. The printed parts are subsequently subjected to washing and sintering processes. The specimens for this study were produced using BPE/ADAM. Details on the approach used are provided in Section 2.1.

It is challenging to select and optimize an AM technique. One difficulty faced is having a reliable part in terms of structural integrity [3]. For a layer-by-layer metal additively manufactured part, the mechanical behavior of the component under various loading directions is influenced by the alignment of the printed layers in a test specimen [14]. It is significant in this regard to study how the layer orientation impacts the properties of the produced item. The test specimens used in this study are cut from BPE/ADAM AM blocks printed in two different directions, as explained in Section 2.3.

It has long been a topic of research to examine the fatigue strength of components, and many test procedures/techniques, life prediction or estimation/calculation methods (stress/strain or energy [15,16,17]), and theories have been established to accomplish this. A growing number of businesses are exploring using AM to improve their designs and production. Consequently, it is essential to examine the fatigue performance of AM components because they might be subject to cyclic loading during application [3,18,19,20]. It is crucial to understand the fatigue behavior of the AM part since fatigue is a major contributing factor to the mechanical failure of many components. The requirement for researching the fatigue behavior of components in high cycles and the time-intensive nature of existing methodologies have led to the development of high-frequency fatigue systems. On the other hand, the development of smaller test specimens is required due to the high cost associated with standard-sized test specimens. In this paper, a small-sized test specimen is designed for bending fatigue testing of BPE/ADAM-made parts in high-cycle regimes. Details are provided in Section 3.

Additively manufactured precipitation hardening (PH) stainless steels can be widely used in industry. Among PH steel alloys, 17-4 PH stainless steel (SS) has comparatively good tensile strength, fracture toughness, and impact strength [14]. The use of 17-4 PH SS has gained increasing attention with the developments in AM [3,14]. Several studies have been conducted on AM-made 17-4 PH SS parts [4,20,21,22,23]. Numerous pieces of research have looked at how post-processing (such as heat treatment) and manufacturing parameters (such as building orientation, laser power, scanning speed, etc.) affect mechanical properties and fatigue performance [20]. For example, Soltani-Tehrani et al. researched how the mechanical properties of PBE 17-4 PH SS pieces were affected by the reuse of metal powder and the position of the printed component on the built plate [4]. Henry et al. studied the mechanical behavior of 17-4 PH SS samples produced using ADAM [21]. They also observed the microstructure of test samples and studied the porosity of the parts [21]. Porro et al. used an artificial neural network to create a prediction model that used microstructural data to estimate the mechanical properties of AM 17-4 PH SS [24]. Suwanpreecha et al. investigated how the printing orientation affected the mechanical, microstructural, and physical properties of 17-4 PH SS test specimens manufactured using the metal-fused deposition modeling approach [22].

To the best knowledge of the authors, there are no studies of the bending fatigue behavior of as-sintered BPE/ADAM components in very high-cycle fatigue (VHCF) regime in the literature. Furthermore, the test specimens used for this study are cut from blocks printed in two different orientations to assess the effect of the print orientation on mechanical performance, specifically fatigue life. In this study, a high-frequency bending fatigue test is carried out on exclusively designed small-sized thin-plate test specimens made of AM 17-4 PH SS blocks. The fatigue life study of the AM 17-4 PH SS test specimens is performed using the stress–life method. The tensile test is also carried out on the specimens produced with two different orientations. The effects of the printing orientation (and the associated large-sized inter-layer/track boundary voids) on the fatigue life and tensile strength of BPE/ADAM as-sintered parts are investigated. The results of this investigation (for ultrasonic bending fatigue testing) are contrasted with those of existing studies to provide a better understanding of the fatigue life of the BPE/ADAM parts and to compare them with those of the wrought material. Nomenclature is listed in Table 1.

## 2. Test Specimen Preparation

The material, specimen design, and preparation steps are described in this section. The bound powder extrusion (BPE) or atomic diffusion additive manufacturing (ADAM) technique is employed to print small 17-4 PH SS blocks. The 17-4 PH SS samples with two different printing orientations are prepared for both bending fatigue testing and uniaxial tensile testing.

### 2.1. Bound Powder Extrusion (BPE)/Atomic Diffusion Additive Manufacturing (ADAM)

For this study, 17-4 PH SS specimens are fabricated by bound powder extrusion (BPE)/atomic diffusion additive manufacturing (ADAM) technology using a Markforged Metal X system. The Markforged Metal X system consists of three main components, namely a Metal X printer (Gen 1), the Wash-1 debinding station, and a Markforged furnace (Sinter-1). During the AM printing process, the bound metal powder filament is extruded to make the green part layer-by-layer. The filament contains wax, polymer binding agents, and metal powder.

**Figure 1:**
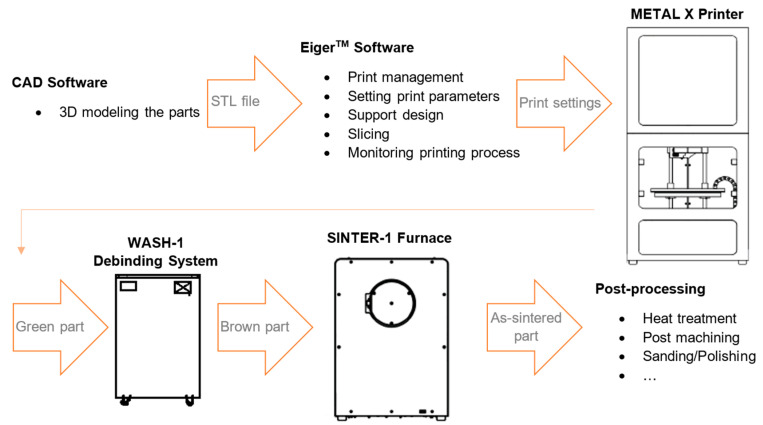
Utilized Markforged bound powder extrusion (BPE) metal 3D printing process [27].

The green part is then washed to remove a portion of the binder [25,26]. The brown part is then placed in a furnace to be sintered, creating a solid as-sintered component. Figure 1 depicts the AM process. The slicing and print setting/management for the Markforged system is performed using Eiger software. The printing settings include the raft, roof/floor layers, wall layers, fill patterns, infill parameters, etc.

**Figure 2:**
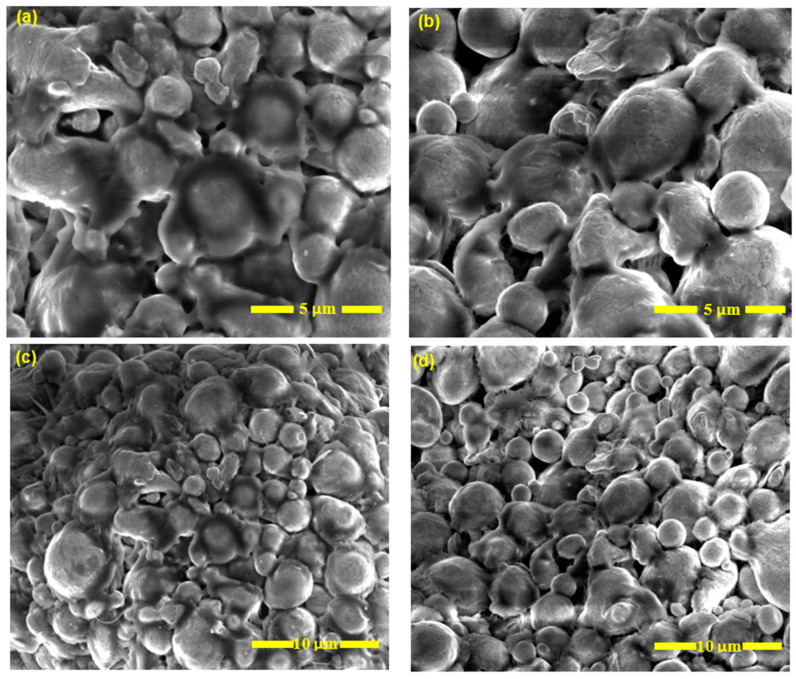
SEM image showing the (**a**,**c**) green part (before debinding/washing) and (**b**,**d**) solvent debound brown part (after debinding/washing).

After printing the “green body” with a Markforged Metal X printer, the WASH-1 was used as the debinding system. During the washing process, while the green body is submerged in the heated Opteon sion solvent in the immersion chamber, the majority of the wax binder is eliminated. The part is moved to the drying chamber once the washing period is complete. It stays for a predetermined period to ensure that any leftover solvent has evaporated into the exhaust system. The approximated immersion and drying times depend on the shape, size, and material used. A thoroughly washed piece that has lost enough mass (about 4.2%) is prepared for the sintering process. This semi-porous part is referred to as the brown part at this point (see Figure 1 and Figure 2). Then, SEM was performed using a Quanta™ 3D DualBeam™ FEG FIB-SEM (Field Electron and Ion Company, FEI, Hillsboro, OR, USA), which is located in the LSU Shared Instrumental Facility. Too much wax binder in a brown part can cause it to fail during sintering, lose quality, or can cause a wax buildup in the furnace. Therefore, the part is weighed and, if necessary, goes through the wash cycle again before being sent for sintering.

The Markforged SINTER-1 tube furnace was used for the sintering process. The brown part is heat-treated under a particular pressure-less heating process in the sintering furnace with the maximum temperature below the melting point. Sintering eliminates the high surface energy of the powder mixture by bonding and diffusing the particles in addition to evaporating the residual binder. During sintering, the brown part transforms into a solid part [28]. Typically, during the sintering process, the brown part shrinks. Therefore, certain factors must be considered while designing the AM parts. Considerations include minimizing stress concentrations at the edges, gradually changing the thicknesses, having well-balanced freestanding elements, and supporting overhangs. After the sintering process is complete, the lightly bonded metal powder of the brown part is transformed into a ready-to-use metal part. The as-sintered piece is removed from the furnace. The supports are then separated. If necessary, post-processing is performed following the requirements. Heat treatment, machining, and sanding/polishing are three frequently used post-processing procedures.

### 2.2. Material’s Composition and Expected Properties

The metal composition of the Markforged 17-4 PH stainless steel filament used for this study and the typical mechanical properties of as-sintered parts are presented in Table 2 and Table 3, respectively. Those data are provided by Markforged [29]. Determination of the chemical composition of the as-sintered part is also available in the literature [22]. No discussion regarding the effects of printing layer orientation on mechanical properties is provided by Markforged.

### 2.3. Test Specimen Preparation with Two Different Printing Orientations

**Figure 3:**
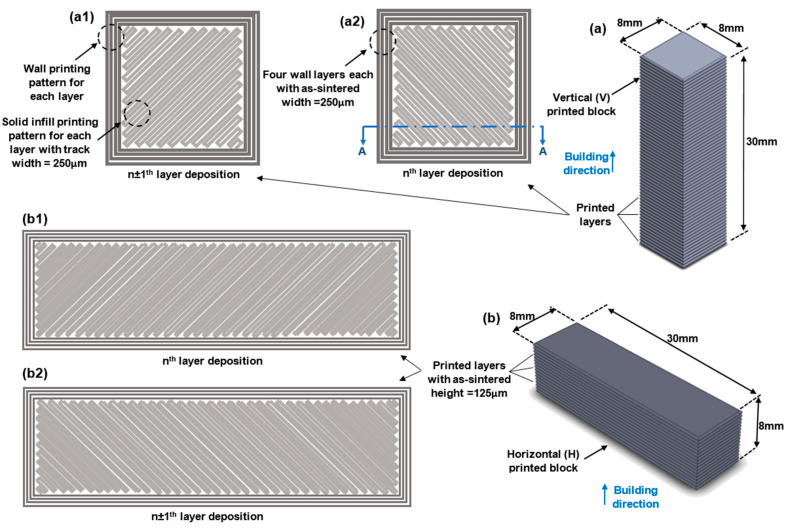
As-sintered 17-4 PH stainless steel (**a**) vertical (V) and (**b**) horizontal (H) blocks using the BPE Markforged Metal X system. (**a1**,**a2**,**b1**,**b2**) Schematic solid infill and wall printing pattern of *n*^th^ and (*n ±* 1)^th^ layers for V and H blocks.

Two sets of 17-4 PH stainless steel blocks were printed using the Markforged Metal X system. One set of horizontally (H)-oriented blocks and one set of vertically (V)-oriented blocks were printed. The blocks have the same outer dimensions, as shown in Figure 3. The printed as-sintered blocks are first cut into thin plates using a wire electrical discharging machine (EDM), followed by the specimens cut from thin plates. The thicknesses of the thin plates are 2 and 0.5 mm for the tensile and fatigue test specimens, respectively. In this fashion, specimens for tensile and ultrasonic bending fatigue tests are both cut from the core of the blocks and, thus, the effects of the two different printing orientations can be studied.

**Figure 4:**
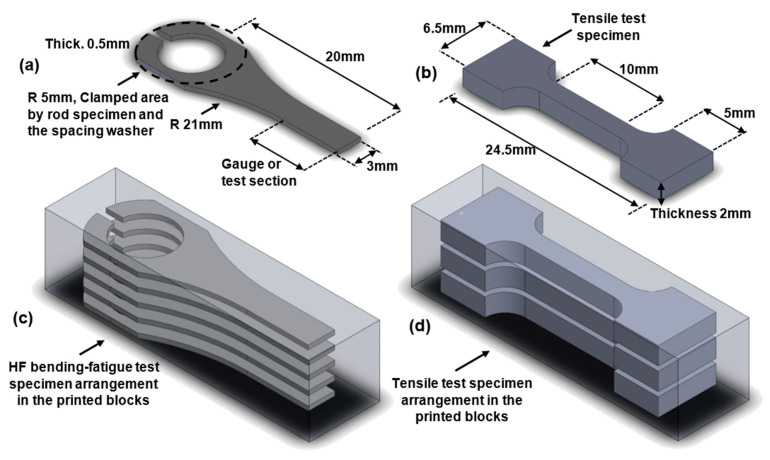
(**c**,**d**) Tensile/ultrasonic bending fatigue test-specimen arrangement in the printed block. (**a**) High-frequency bending fatigue test specimen’s dimensions. (**b**) Tensile test specimen dimensions.

**Figure 5:**
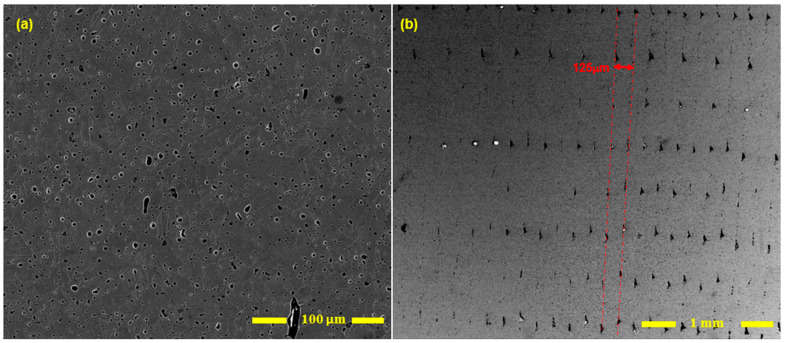
SEM images of the cross-section (Section A-A, Figure 3) of the polished and etched as-sintered part (V blocks, see Figure 4). (**a**) Voids in component and (**b**) voids at the layers’ interfaces (layers are in the shown direction, and the post-sintering layer thickness is 125 μm). Note—the building direction is along the red arrow shown in (**b**).

Figure 3 depicts a schematic of the infill printing patterns (filament laying patterns) for V and H blocks. The tested specimens have the two distinct infill patterns. The following sections provide more information on the implications of such a filament-laying arrangement. Figure 4 shows how the test specimens are arranged within the printed AM 17-4 PH SS blocks. As the samples are cut from the infill region of the as-sintered blocks, the effects of two different layer orientations will be examined. The specimens cut from the vertical (V) and horizontal (H) blocks were marked with the letters V and H, respectively. Figure 5 displays an SEM image of the cross-section of a V block which shows the printed layers and the filament laying pattern.

A smooth and mirror-finished surface is required for both sides of the ultrasonic bending fatigue test specimens. After cutting the test specimens, first they were polished using a single-wheel metallographic hand polisher. Then, the surfaces of the specimens were mirror-finished using a vibratory polisher. Before testing, the specimens’ thicknesses were measured at their gauge sections to calculate the induced stresses and verify uniform thickness throughout the samples [30].

### 2.4. Ultrasonic Bending-Fatigue Test

**Figure 6:**
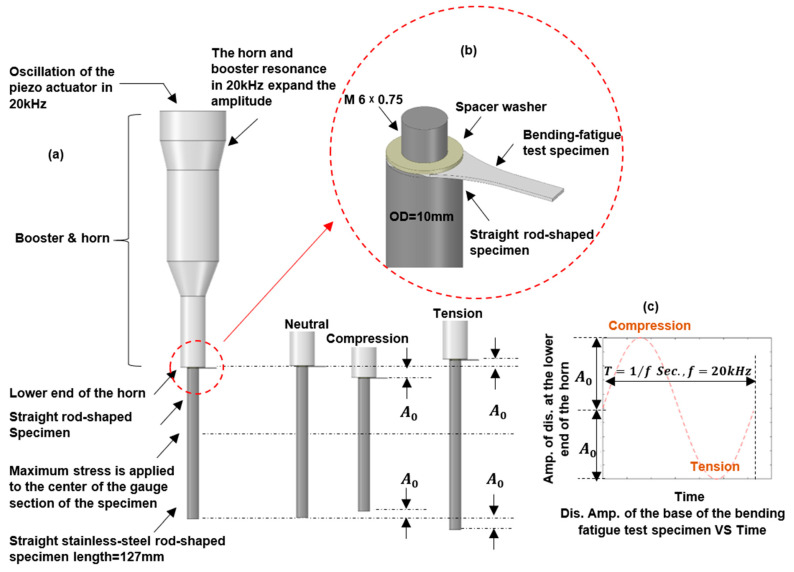
(**a**,**c**) The ultrasonic fatigue system operation principle and bending fatigue testing arrangement; (**b**) test arrangement [30].

An ultrasonic fatigue testing system was used to carry out the bending fatigue test (SHIMADZU USF-2000A, Kyoto, Japan). The USF-2000A generates longitudinal tension–compression displacement in a test specimen at a frequency of 20 kHz [30]. The designed test specimen for this study (ultrasonic bending fatigue testing) is situated between the top surface of the straight rod-shaped sample and the bottom (lower) end of the horn (see Figure 6). Detailed considerations for ultrasonic bending fatigue specimen design are presented in Section 3.

### 2.5. Tensile Test

Uniaxial tensile tests were performed on the V and H test specimens with a constant strain rate of 8.3 × 10^−4^/s using an MTS Alliance RF/100 tester (MTS Systems, Eden Prairie, MN, USA). Multi-digital image correlation (Multi-DIC or 3D-DIC [31]) equipped with software MultiDIC (MIT, Cambridge, MA, USA) and cameras (XIMEA GmbH, Münster, Germany), a non-contact optical-numerical approach, was utilized to measure the change in the specimen’s length throughout the tensile test and to calculate the strain.

## 3. Ultrasonic Bending-Fatigue Test Specimen Design

The ultrasonic bending fatigue test specimen deflects throughout its length when the supported end vibrates in a vertical direction. The gauge section experiences the maximum bending stress due to the test specimen’s deflection shape. Ghadimi et al. presented a general design approach for bending fatigue test specimens [30]. The specimen design needs to be assessed and validated for different testing materials. Consequently, the specimen design is discussed in brief in this paper. A cantilevered beam with an amplitude of displacement of $A_{0}$ at the fixed end is used to model and solve the vibration response of the test specimen. The dynamic Euler–Bernoulli equation for a homogeneous beam with a uniform cross-section area over the length of the beam is implemented to investigate the deflections and stress distribution of the given beam. The problem is solved using Python code in Jupyter Notebook. The properties for 17-4 PH stainless steel are used for the calculations.

The oscillatory system includes the horn, booster, straight-rod-shaped specimen, and bending fatigue specimen. To resonate at 20 kHz, the system’s natural frequency should be within an acceptable range. To prevent areas of stress concentration and to ensure that the specimen breaks from the designated gauge section, it is also necessary to evaluate the specimen’s vibration shape, bending mode, and stress distribution throughout its length.

Ghadimi et al. first presented the design for the ultrasonic bending fatigue test specimen used in this study [30]. They developed an analytical solution in MATLAB and performed finite element analysis (FEA) simulation in ANSYS to optimize the specimen design. The specimen’s failure and deflection shape were also investigated through experiments, and the test outcomes were compared with the calculations obtained from the theoretical solution and also the FEA simulation. The comparisons showed that the specimen’s responses are desirable during testing [30].

As the testing material is different for this study, the specimen design needs to be verified for the natural frequency, vibration shape, and strain/stress distribution to ensure the design works for 17-4 PH stainless steel. As such, the analytical solution introduced in the Ghadimi et al. paper [30] was used when considering the requirements of the current study. The web-based interactive computing platform Jupyter Notebook and the Python programming language were used to solve the governing equation and obtain the results. Furthermore, the FEA simulation was carried out in the ANSYS 2022 R1 software package to verify the design and better understand the stress/strain distribution.

### 3.1. Vibration Response Theoretical Analysis

A cantilevered beam with two prismatic portions subjected to support excitation in the absence of external loadings is used to model and solve the bending fatigue test specimen’s vibration response. The cantilever beam is dynamically excited by the applied reciprocal motion at the fixed end of the beam. The frequency of the displacement is 20 kHz. The displacement amplitude is $A_{0}$ which is the amplitude at the horn’s lower face. The forced vibration features of the given beam then are solved using the dynamic Euler–Bernoulli equation.

The y axis is specified along the applied displacement, while the x axis direction is determined along the beam. The beam’s deflection in the direction is $w\left(x,t\right)$.

The modeled beam is homogeneous, and the cross-section area is the same along its length. Consequently, E, I, and A are independent of the beam’s location. Applying an external time-varying distributed force of $f\left(x,t\right)$ in the y direction, the following fourth-order partial differential equation (PDE) of the dynamic Euler–Bernoulli equation is applicable to obtain the transverse displacement of the beam:

(1) $$EI\frac{\partial^{4}w\left(x,t\right)}{\partial x^{4}}+\rho A\frac{\sigma^{2}w\left(x,t\right)}{\sigma t^{2}}=f\left(x,t\right)$$

where ρ is the material’s density, t represents time, and the expression $\frac{\partial^{2\;}w\left(x,t\right)}{\partial t^{2}}$ is the body’s y-direction acceleration. Furthermore, EI stands for the flexural rigidity, where E is the elasticity modulus, while I is the second moment of inertia of the beam’s cross-section area (A).

In the context of this research, there are no external forces present. Hence $f\left(x,t\right)$ is 0. An alternative version of Equation (1) is created when $w\left(x,t\right)=W\left(x\right)T\left(t\right)$ based on the variable separation, where $c=\sqrt{\frac{EI}{\rho A}}$ and ω represent the angular velocity. Equation (2) is as follows:

(2) $$\frac{c^{2}}{W\left(x\right)}\frac{d^{4}W\left(x\right)}{dx^{4}}=-\frac{1}{T\left(t\right)}\frac{d^{2}T\left(t\right)}{dt^{2}}=\omega^{2}$$

$W\left(x\right)=Ce^{sx}$, where C is a constant, is presented as the solution to the $W\left(x\right)$ equation. (By solving the equation, s is ±β or ±iβ) and the solution’s form for the $W\left(x\right)$ equation may be expressed as follows, where $C_{1}$ to $C_{4}$ are constants derived by applying the boundary conditions. Additionally, the proposed $T\left(t\right)$ equation’s solution is shown below. Equations (3) and (4) are as follows:

(3) $$W\left(x\right)=C_{1}\cos\beta x+C_{2}\sin\beta x+C_{3}\cosh\beta x+C_{4}\sinh\beta x$$

(4) $$T\left(t\right)=A\cos\omega t+B\sin\omega t$$

Separate equations were solved for each of the two beam parts. The corresponding constants were calculated by considering the four continuity conditions at the point where the cross-section changes and the two boundary conditions (BC) at each of the free and the fixed ends. The suitable sinusoidal term corresponding to the horn’s end amplitude describes the BC for the beam’s clamped end. Furthermore, the slope is zero at this point. The beam’s free end has a zero bending moment and shear force. Continuity conditions include equivalence of the beam’s slope, internal shear forces and bending moments, and the vertical displacements of parts 1 and 2 of the beam at the connection location. With the assumptions above, the $W\left(x\right)$ equation is resolved. When the oscillations at time *t* = 0 and time when $\omega t=\frac{\pi }{2}$ are taken into account, $T\left(t\right)=\sin\omega t$ is obtained as a solution for Equation (4), and, finally, $w\left(x,t\right)=W\left(x\right)T\left(t\right)$ is explained.

By solving the related eigenvalue equation, the natural frequencies of the undamped system with no external forces were determined. Solving the beam’s deflection equation at each location along the beam, the internal bending moment and, consequently, the normal stress, were also calculated. The maximum values for both internal bending moment and deflection occur in the same location of the beam where the maximum normal stress is located. If the excitation vibration’s amplitude is altered, similar vibration shapes/forms with various vertical deflections are generated. Therefore, changing the excitation amplitude may produce different maximum normal stress values at the gauge section.

**Figure 7:**
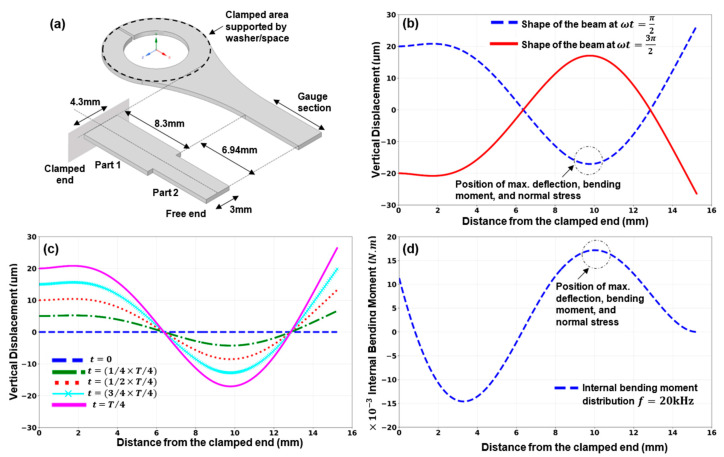
(**a**) The modeled cantilever beam. (**b**) The beam’s shape at $\omega t=\frac{\pi }{2}\;and\;\frac{3\pi }{2}$. (**c**) The beam’s shape from time t=0 to $t=\frac{T}{4}$ for $A_{0}=20\;\mu m$, f=20 kHz, and (**d**) the internal bending moment distribution.

Figure 7 shows the specimen shape for a particular horn’s tip displacement at instances when $\omega t=\frac{\pi }{2}$ and $\omega t=\frac{3\pi }{2}$. The change in the test specimen’s profile between times t=0 through $t=\frac{T}{4}$ (T represents the period of cycles), is also shown in Figure 7.

### 3.2. Finite Element Analysis (FEA)

The fatigue tester’s applied acoustic wave ultimately produces cyclic displacement at a frequency of 20 kHz delivered to the test specimen’s clamped end. The “8-node quadratic SOLID185” elements were used to mesh the geometry of the test specimens, and a mesh analysis was performed for FEA simulation. First, a modal analysis was conducted to study the mode shapes and natural frequencies. The stress/strain distribution was then examined using a harmonic analysis with the specified cyclic displacement amplitudes and 20 kHz frequency.

**Figure 8:**
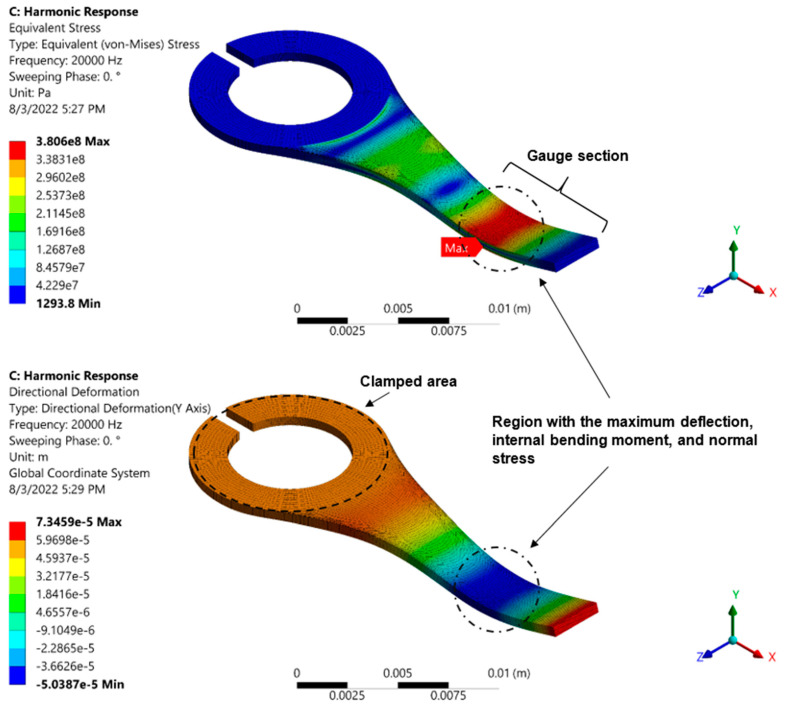
FEA simulation results for 20 kHz and displacement amplitude of 55 μm. (**top**) Shows the corresponding stress distribution and the specimen’s mode shape and (**bottom**) the strain distribution (in the Y direction).

The design displays the intended and appropriate bending mode shape at the system testing frequency based on the findings from the finite element analysis. The displacement distribution throughout the specimen’s length as well as the equivalent von Mises stress distribution is shown in Figure 8. The maximum bending deflection and highest normal stress value occur at the mode shape’s peak. The outcomes of the theoretical analysis and the FEA simulation show good agreement.

## 4. Results and Discussion

### 4.1. Tensile Test Results

**Figure 9:**
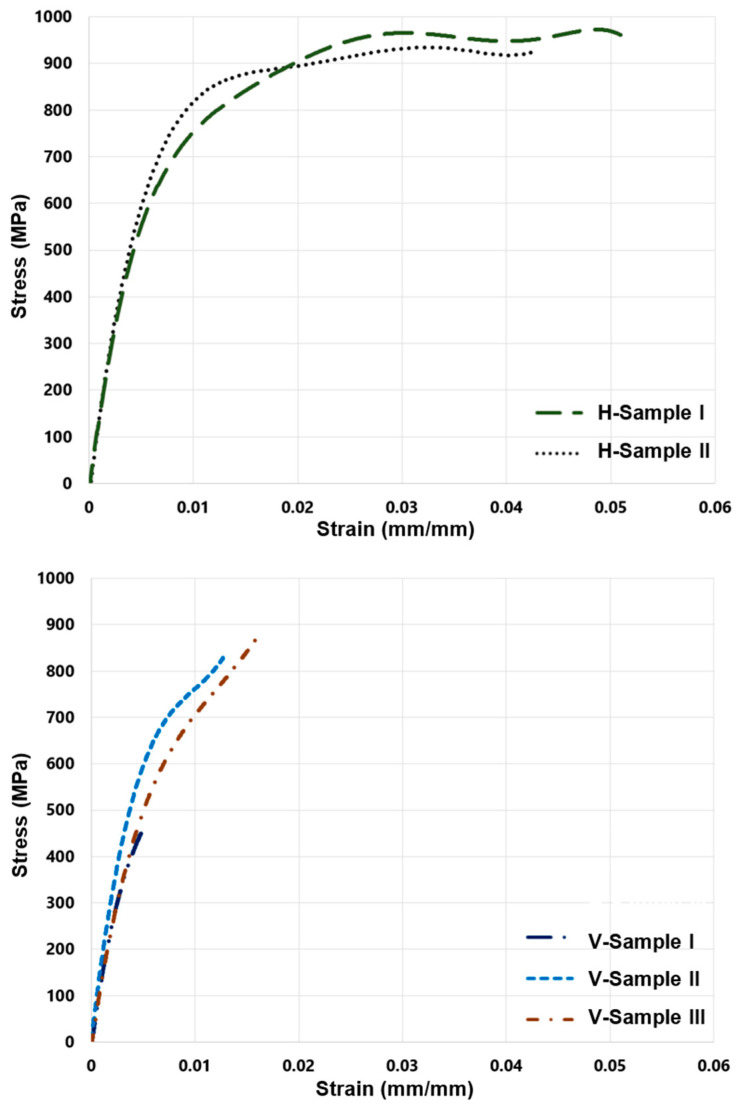
Tensile test results for the H and V test specimens.

The V and H test specimens underwent a uniaxial mechanical tensile test. Multi-digital image correlation (Multi-DIC or 3D-DIC) was utilized to measure the change in the specimen’s length throughout the tensile test for strain calculation. Measurements were made of the applied load, the variation in the test piece’s length, and the engineering stress and strains were calculated accordingly. The results of the tensile tests for H specimens and V specimens are shown in Figure 9. Engineering strain ($\varepsilon_{e}$) is in the horizontal axis of the stress–strain curve, while engineering stress ($\sigma_{e}$) is on the vertical axis. The stress–strain curve has elastic and plastic regions. The ultimate tensile strength ($\sigma_{uts}$) of the material is the maximum stress when the necking starts. The test piece’s cross-section area rapidly shrinks during the necking process.

The test specimens’ ductility, which represents the capacity to flex plastically before breaking, is compared against each other by comparing their fracture strain. Material is more ductile when it has undergone large plastic deformation before fracture. Toughness refers to a material’s capacity to absorb energy up to fracture, which is measured by calculating the area below the stress–strain curve. According to the reported tensile test outcomes, the H specimens are significantly more ductile and tougher than the V specimens (see Figure 9).

**Figure 10:**
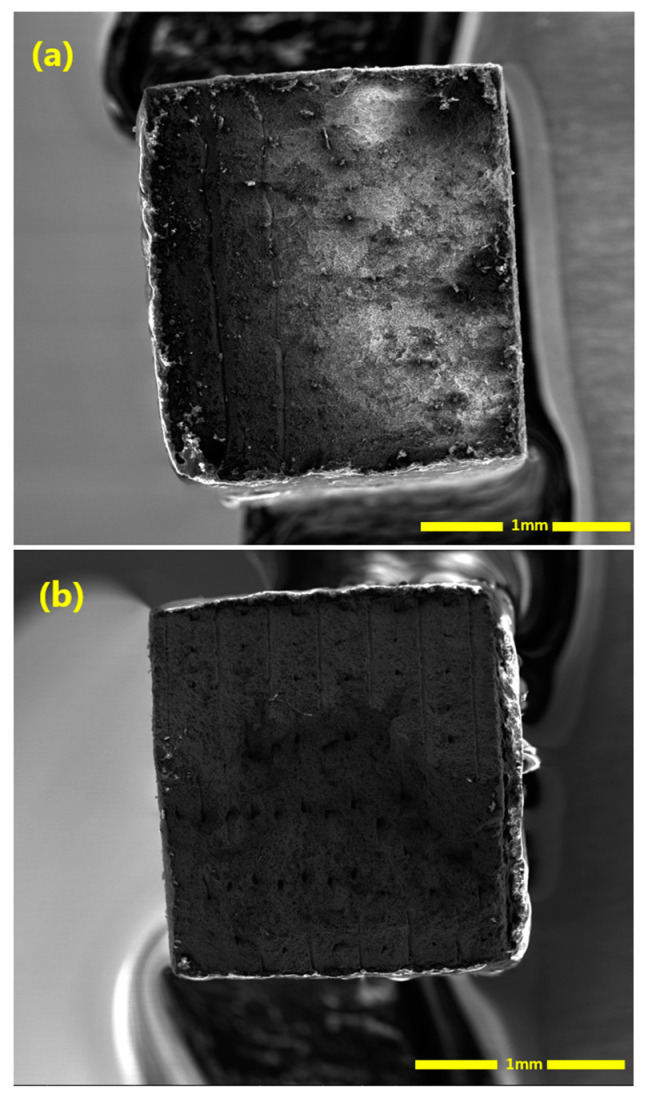
SEM image showing the fracture surfaces of the specimens. (**a**) Fracture surface of the vertically-printed specimen; (**b**) fracture surface of the horizontally-printed specimen.

The V specimens indicate a high density of uneven protrusions on the fractured surface (see Figure 10a). As the layer orientation is perpendicular to the tensile load direction, large pores in-between printing tracks are in the plane of the fractured surface. The uneven features are remnants of sintered parts around those large pores. In Figure 5b, it is observed that large numbers of pores exist along the print tracks/layers. Moreover, the pores are typically strip-shaped, with long axes along the print-layer boundaries. For the V specimens, the tension load is perpendicular to the print layers. This makes V specimens prone to Mode I fracture. However, for the H specimen case, the tensile stress is parallel to each fabrication layer, which reduces the influence of the pores observed along the track/layer boundaries on the mechanical properties. Therefore, the strip-shaped pores along the layer boundaries are the critical reason why significantly inferior plasticity is obtained for V specimens. The H specimens have those strip-shaped pores along the loading direction and, thus, are more visible in Figure 10b. As these strip-shaped pores are in the parallel plane to the tensile load direction, they are less likely to act as strong and immediate stress-raisers—in contrast to the V specimens. Therefore, the printing layer orientation determines the tensile behaviors of the specimens. It is simpler for cracks to form and merge in the V tensile test specimens because the weak interfacial layers lie perpendicular to the direction of the tensile force. However, because the printing layers are parallel to the loading axis in the H test samples, the formation of cracks are delayed. The H specimens indicate higher plasticity than the V specimens. However, compared with typical wrought counterparts, the strain-to-failure value is still low. Apart from the small-sized sintering defects between powders, with careful observation in Figure 5b, it seems that the large strip-shaped pores along the printing track/layer boundaries also extended slightly to the track boundaries within the same fabrication layer, which deteriorates the ductility of H specimens.

### 4.2. Ultrasonic Bending-Fatigue Test Results

**Figure 11:**
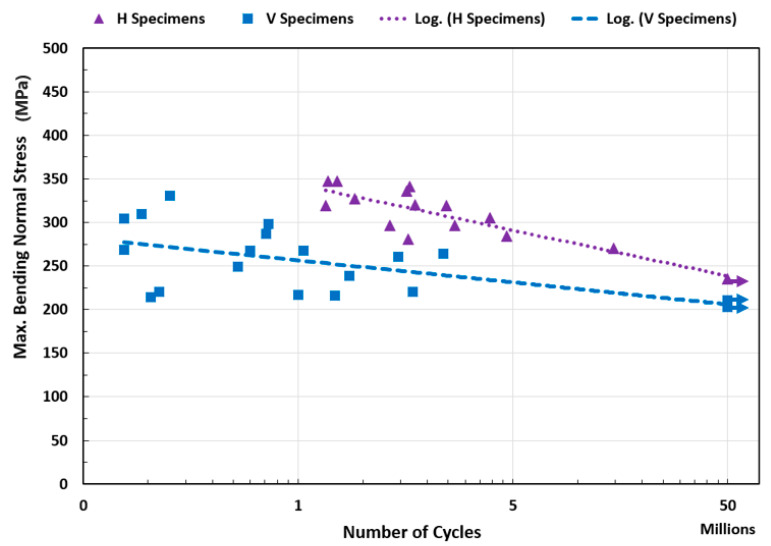
Ultrasonic bending fatigue test results (stress–life best fit curve) for H and V specimens.

A constant amplitude strain-controlled, fully-reversed (R=−1) bending fatigue test was carried out for V and H specially-designed test specimens in a high-frequency regime. Each specimen’s clamped end vibration amplitudes were measured, and the maximum applied normal bending stress at the gauge section was calculated. The test findings are presented in the stress–life graph in Figure 11.

Based on the study’s findings in Figure 11, the printing orientation significantly affects the parts’ fatigue life. It has a tremendous effect on the fatigue behavior of the piece. The H specimens show significantly higher fatigue strengths than the V specimens.

### 4.3. Discussions and Fractography

**Figure 12:**
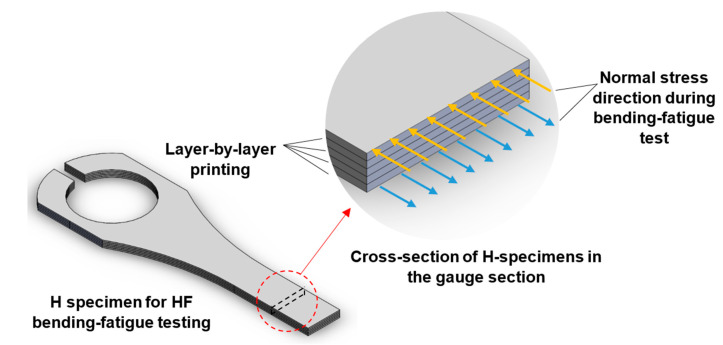
Applied normal stress direction in the cross-section of the H specimens during ultrasonic bending fatigue testing.

The BPE 3D-printing technology’s layer-by-layer construction results in parts with transverse isotropic material characteristics. Otherwise, the orientations of the printing layers affect the component strength in the loading direction. For these transverse isotropic materials, one set of properties along one axis differs from planes normal to that axis. The normal stress applied during the bending fatigue and tensile tests is parallel to the printed layer for the H specimens and perpendicular to the printed layer for the V specimens. Consequently, H specimens have higher strength and elongation before failure in the applied load direction during the fatigue and tensile tests (see Figure 9 and Figure 11). Based on the findings of the tensile test, the V specimens behave more like brittle material than the H specimens. Therefore, H specimens can have plastic zones that are relatively larger. This allows the H specimens to tolerate an increased stress field around defects and prevents cracking when being loaded (see Figure 12).

**Figure 13:**
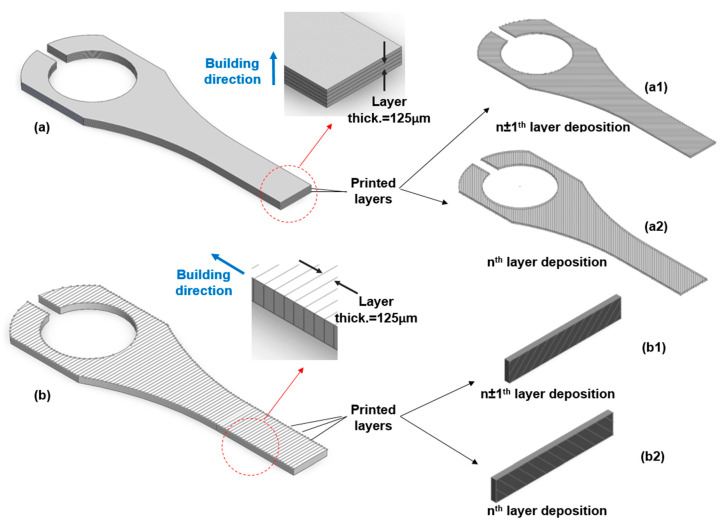
Schematic solid infill printing pattern of *n*^th^ and (*n* ± 1)^th^ layers for H (**a**,**a1**,**a2**) and V (**b**,**b1**,**b2**) ultrasonic bending fatigue test specimens.

The as-sintered printed block utilized for this study has a layer thickness of 125 μm and is constructed layer-by-layer. The nozzle lays a 250 μm width track of softened filament material to form each layer, following the pattern for the wall and infill section (see Figure 3 and Figure 13). Four 250 μm width wall layers are laid in the building direction (total wall thickness is 1 mm). While the wall laying pattern is the same for all the layers, the solid infill printing pattern for the *n*^t^^h^ layer differs from the pattern of the (*n* ± 1)^th^ layer (see Figure 3 and Figure 13). In the infill area, the material tracks are placed perpendicular to the direction of the underneath layer’s track. In contrast, the wall layers are stacked on top of one another, with the tracks running in the same direction.

**Figure 14:**
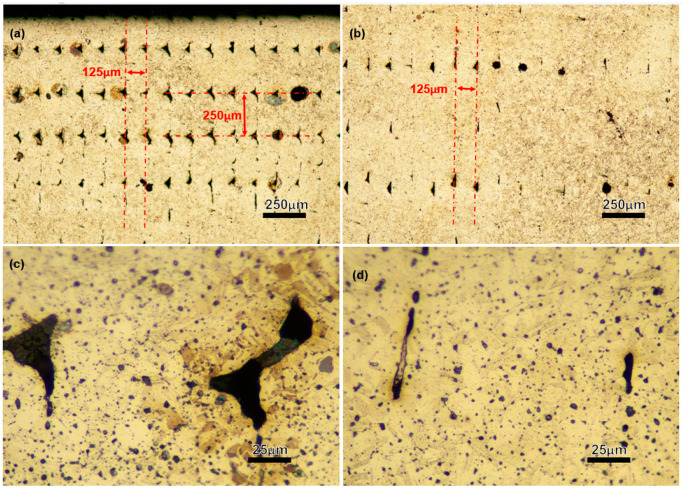
For a polished and etched surface of an as-sintered V block’s cross-section (Section A-A Figure 3), layer interface/track boundary voids at the (**a**,**c**) wall region and (**b**,**d**) the infill region and (**d**) pores/voids at the infill/main part region (the building direction is along the red arrow shown in (**b**)).

The metal powders bond together during the sintering process to produce a solid part, but voids remain in the layer interfacial and track boundary regions, forming layer defects. Pores are extended in the track direction at the interface of the printed tracks. The wall section also has layer defect voids in both the layer interfacial and track boundary regions. Due to the laying pattern applied for the walls, the voids (layer defects) in this region are extended in both the layer’s laying and in the part’s building directions. The layer interfacial/track boundary voids for the wall and the infill regions are shown in Figure 3 and Figure 14. Because the gauge section of the test specimens does not include the wall region of the printed block, only the infill layer interfacial/track boundary voids are present in the gauge section of this study’s test specimens (tensile and ultrasonic bending fatigue).

Voids/porosities affect the part’s mechanical performance [3,20,32,33]. The shape (irregular-shaped, spherical, sharp corners, etc.), size, and distribution of voids can be essential factors in the mechanical behavior study of the AM parts [14]. Geometry, size, and the distribution of the voids can be affected by the heat treatment received in the sintering process. The part’s size, geometry, and printing parameters also affect the voids’ geometry, size, and distribution.

The printed components contain two different types of voids/pores. The larger one is the layer interfacial/track boundary voids discussed above. Additionally, the relatively small-sized porosities are the result of trapped gases of residual binders inside the piece or the pores left from metal particles which are not fully densified. Given that there are pores inside the component (see Figure 5, Figure 10, and Figure 14), it shows that complete densification did not occur during the sintering process, meaning that the pores’ spaces were not eliminated. However, residual pores are not the only explanation for the voids present. The brown part may still contain residual binder left over from the solvent debinding process, and also some binder that will be removed in the thermal debinding during the sintering process. The evaporated binders may become caught inside the pieces and cause porosities inside (see Figure 5 and Figure 14). The relative density for BPE/ADAM as-sintered blocks is 96%, as shown in Table 3. Wrought 17-4 SS (H-900) has a reported density of 7.8057 (g/cm^3^) [34]. The less-dense as-sintered BPE/ADAM further supports the presence of pores and illustrates the existence of voids in the components.

**Figure 15:**
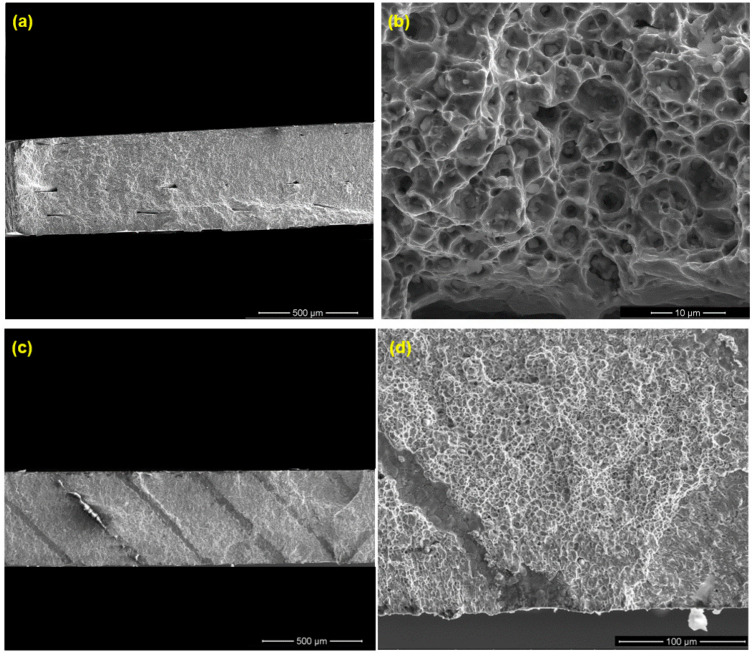
SEM image of the fracture surfaces after ultrasonic bending fatigue testing for (**a**,**b**) H and (**c**,**d**) V test specimens. (**a**,**c**,**d**) Layer interfacial/track boundary voids at the fracture surfaces; (**b**) fracture surface of as-sintered parts showing small-sized defects (pores and inclusions).

Unlike the large pores, the small-sized pores are uniformly distributed, which are not strongly linked with the printing orientation. However, the large pores, as discussed above, are strip-shaped, mainly extending along the fabrication layer direction and slightly extending to the track boundaries within the same fabrication layer. Considering that, during the fatigue test, the tension stress in the V specimens is vertical to the fabrication layers, a crack is easier to initiate along the grain boundaries. This can be one of the reasons why lower fatigue life is obtained for the V specimens (Figure 11). Figure 15 shows the fracture surfaces after the fatigue test. It is clear that the V specimens fracture along the layer boundaries, which is evidenced by the grooves, which are most likely the pores along the layer boundaries observed in Figure 14a. This verifies the discussion above that the cracks preferably initiate and propagate along the fabrication layer boundaries. Figure 15b illustrates small-size defects (pores and inclusions) that are present throughout the as-sintered components. Based on Suwanpreecha et al., two phases of micron-scale inclusion are present in the as-sintered parts [22]. The first is a Si- and O-rich phase, reported as SiO_2_ and the second is a Mn-, Cr-, and O-rich phase, which is defined as MnCr_2_O_4_ [22].

Furthermore, in the high-cycle fatigue (HCF), the crack initiation/formation phase dominates the total fatigue lifetime [14]. Internal voids create local stresses that, when subjected to cyclic loading, result in the creation of local plastic deformations and, ultimately, the onset of fatigue cracks. The crack grows in size through loading cycles, and the test piece fractures consequently. The surface of the test specimen experiences the highest normal stress delivered during bending fatigue loading (test specimens were meticulously polished to remove flaws and decrease surface roughness). Therefore, the fracture initiation is more influenced by defects closer to the surface. The gauge section defects closer to the specimen surface may significantly impact the crack initiation when considering the stress distribution in the specimen cross-section during the bending fatigue test. In this regard, the location of the defects may also have a significant role in crack initiation and propagation. Based on the findings of this study (see Figure 11), specimens (of the same printing orientation) that are tested with relatively close maximum normal stress values may not always experience the same fatigue life. This is explained by the components having voids, pores, impurities, or flaws not evenly distributed nor in the same size/shape. The defects might vary in size, shape, and distribution, which justifies the data’s dispersion.

The fracture surface after the ultrasonic bending fatigue test shows two different regions, namely a smoother area that shows steady fatigue crack propagation and a rougher part created by the ultimate fracture. The fracture type of the ultrasonic bending fatigue test specimens may be evaluated by observing the fracture surfaces; it combines trans- and intergranular fractures. The river markings and significant area fractions of cleaved facets indicate a brittle fracture. At the fracture surface, a grain boundary fracture is visible. These are also regions with tortuous features.

Crack initiation, crack growth or propagation, and the final ruptured areas may all be seen on the fracture surface. The material’s distribution of defects suggests that a fracture surface may have several crack initiation sites. As a result, when propagating, the cracks caused by various defects may interact and coalesce. Before the final fracture happens, initiated cracks grow to a critical size. Varied specimens experience different crack growth rates because the loads and imperfections for each test piece are different. Due to the crack’s relatively shorter growth time before the ultimate fracture, the ratio of the crack growth area to the fracture area decreases for higher loading levels.

**Figure 16:**
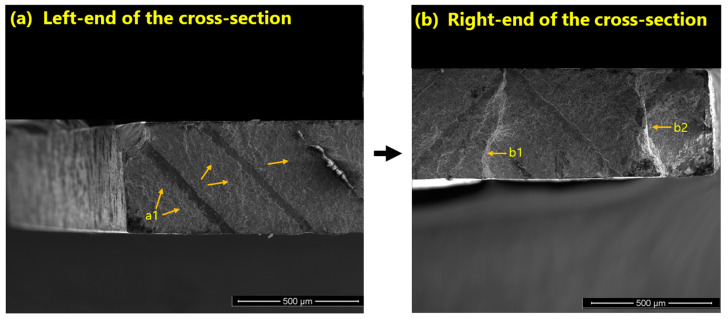
(**a**) SEM image of the fracture surface of a V fatigue specimen. The initiation of a crack from a pore close to the surface corner in region (a1), and (**b**) the crack propagation until it reaches the right end of the cross-section and tears off; the attached leftovers of the cross-section tear off in the two stages of (b1) and (b2).

In the case of the vertical specimens, the crack initiated from the left corner and propagated through the cross-section until it reached a print line (see Figure 16). At this point, it grew sideways and tore off a small section at the opposing corner. Subsequently, the growth direction straightened up along the width of the fracture section until the specimen failed.

**Figure 17:**
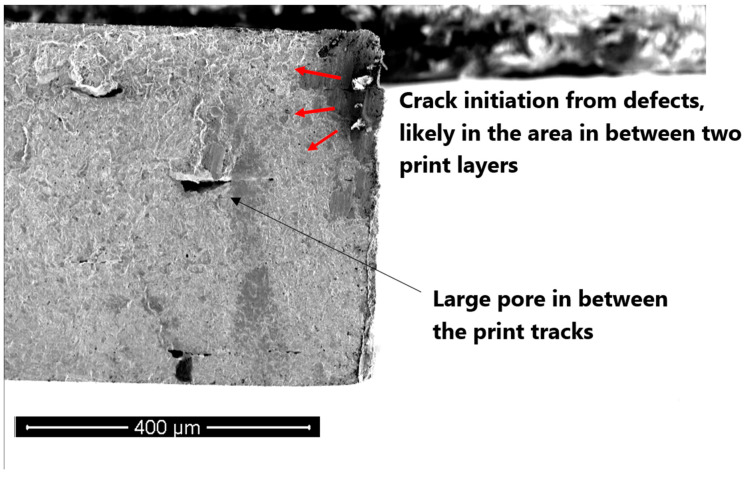
SEM image of the fracture surface of the H fatigue sample. The initiation of crack from sintering defects.

In the case of horizontal samples—in one case— a crack starts from a sintering defect and grows across the cross-section until total failure occurs (see Figure 17). The defect is likely to have been formed close to the print layer.

In general, in both of the cases, initiation seems to have been started from stress-raisers, such as sintering defects or pores, although, as discussed, the H samples endure more plasticity as the orientation of pores in them is such that makes them more resistant to axial loads and stresses. Specifically, though the pores did extend along the track boundaries within the same fabrication layer (vertical to the tension in H specimens), the pores mainly extend within the inter-layer boundaries (parallel to the tension stress direction). Therefore, compared with the V counterparts, the H specimens show higher ductility.

### 4.4. In Comparison with the Literature Data

**Figure 18:**
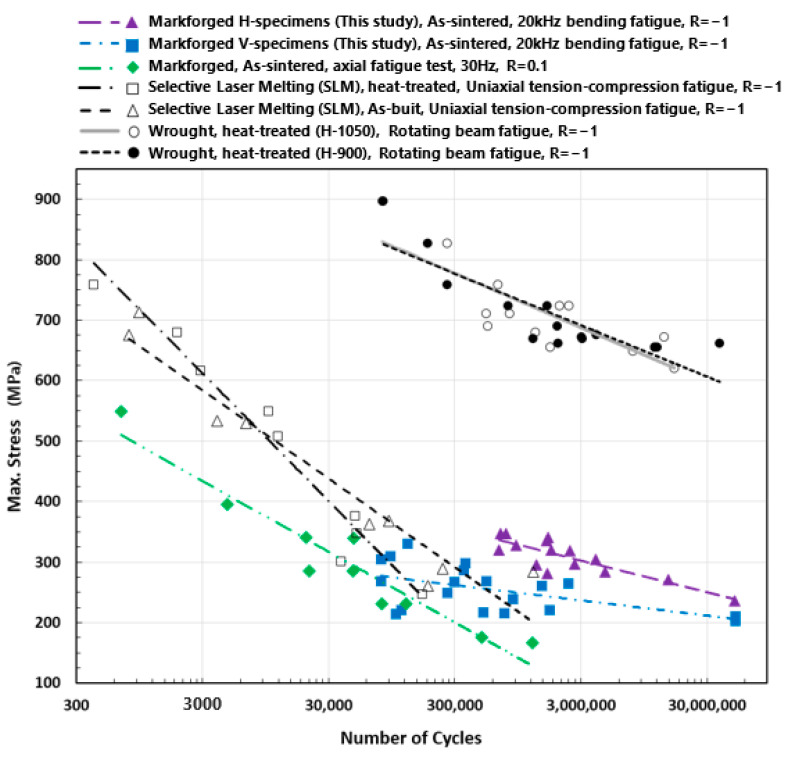
Comparing the stress–life data of a 17-4 PH SS test specimen made of wrought material [35] (rotating-beam fatigue test) with those of test specimens fabricated using Markforged BPE (HF bending-fatigue test, as used in this study, and axial fatigue test [36]) and SLM (uniaxial tension–compression fatigue test) [37].

A part’s fatigue life varies based on the geometry, size, testing method, testing procedure, etc. Consequently, it is challenging to directly compare the findings of this study to the body of literature. However, comparing the findings of the fatigue life studies contributes to a better understanding of the subject in general. Figure 18 contrasts this study’s findings with stress-related data from the literature. To compare the life of wrought with additively manufactured components, the rotating beam fatigue properties of wrought 17-4 PH SS (H-900 and H-1050) are presented [35]. Kedziora et al. investigated the fatigue life of test specimens made of 17-4 PH SS and produced by the Markforged Metal X machine (similar to the AM method/material used in this study) [36]. They conducted an axial fatigue test with an R = 0.1 stress ratio and a constant 30 Hz testing frequency. Kedziora et al. conducted their fatigue test on a test specimen of a typical size. Using the Metal X printer, they 3D fabricated the full-size specimens with all features (instead of printing a block and cutting test specimens out of it). As a result, in addition to the inter-layer and layer boundary defects of the main internal portion, their test specimens also contain the wall layers and their intrinsic faults (see Figure 3). Although the fatigue life of test specimens can vary depending on the test methods, specimen design, size, and other factors, the presence of wall layers can also have an impact. This study’s findings are reasonable and provide insight into higher cycle regimes.

In terms of size and underlying causes, the defects found in the examined BPE components may be divided into two groups. First, there are larger defects/voids that are present in the inter-layer area and at layer boundaries. The second type of void is smaller in size and is caused by trapped gases or un-fully densified powders during the sintering process. Both categories of defects have an impact on fatigue life and cause the material to last for less time than a wrought material. The results of this work are contrasted against the life reported for a 17-4 PH SS component manufactured using SLM technology to approximately estimate the influence of each type of defect on the fatigue life of the parts. As-built and heat-treated 17-4 PH SS SLM components were examined for uniaxial tension–compression fatigue life by Yadollahi et al. [37]. They concluded that the test specimen’s lifespan is significantly impacted by the pores (which are typically caused by a lack of fusion or keyholes) present in the AM components. The type of defect reported in their research is comparable to the large-sized pores seen in the BPE components of this investigation. Therefore, it may be concluded that if the same test is conducted on similar specimens manufactured using SLM and BPE, the inter-layer/layer boundary defects identified in the BPE portions may result in reduced fatigue life.

## 5. Summary and Conclusions

In this study, microstructure evaluation, uniaxial tensile tests, and ultrasonic bending fatigue tests were performed on 17-4 PH stainless steel produced with extrusion-based bound powder extrusion (BPE)/atomic diffusion additive manufacturing (ADAM) technology in two different orientations, namely vertical and horizontal. The following conclusion can be reached:

(1) Two types of defects were observed inside the samples after sintering. First, large-sized pores originated from a lack of sintering mainly extended along the fabrication layer boundaries and slightly went to the track boundaries within the same fabrication layer. Second, the small-sized pores stemmed from trapped gas and residual binders that show shapes close to spherical or un-densified metal powders. Overall, the large defects show a more significant effect on the mechanical property of the specimens;
(2) The vertically-oriented specimens show inferior ductility and toughness than that of the horizontally-oriented counterparts, which is ascribed to the large pores (strip-shaped) extending along the fabrication tracks/layer boundaries;
(3) Similarly, vertically-oriented specimens have a lower fatigue life than the horizontally-oriented parts, which also stemmed from the large defects. Compared with wrought 17-4 PH alloy, lower ductility was observed in the horizontally-oriented specimens in this study, which is due to the existence of both small defects and large defects.

## Figures and Tables

**Table 1:** Nomenclature.

$A\left(x\right),\;A$	Cross-Section Area ($m^{2}$)	T	Period (sec)
$A_{0}$	Horn tip’s displacement amplitude (m)	$V\left(x,t\right),\;V$	Internal shear force (N)
E	Modulus of elasticity (Pa)	$w\left(x,t\right)$	Deflection of the beam in y direction (m)
f	Frequency (Hz)	x	Global x coordinate (m)
$f\left(x\right)$	Applied external distributed force (N)	$x_{1}$	X coordinate for beam Part1 (m)
$I\left(x\right),\;I$	Second moment of inertia of the cross-section area ($m^{4}$)	$x_{2}$	X coordinate for beam Part2 (m)
L	Total length of the beam (m)	y	Global y coordinate (m)
$L_{1}$	Length of Part1 (m)	z	Global z coordinate (m)
$L_{2}$	Length of Part2 (m)	ρ	Density (kgm3)
$M\left(x,t\right),\;M$	Internal bending moment (N.m)	σ	Normal stress (MPa)
t	Time (sec)	ω	Angular frequency (radsec)

**Table 2:** Markforged 17-4 PH stainless steel composition [29].

Composition	Chromium	Nickel	Copper	Silicon	Manganese	Niobium	Carbon	Phosphorous	Sulfur	Iron
Amount	15–17.5%	3–5%	3–5%	1% max	1% max	0.15–0.45%	0.07% max	0.04% max	0.03% max	bal.

**Table 3:** Markforged 17-4 PH stainless steel mechanical properties [29].

Mechanical Property	Standard	Values for As-Sintered Parts	Unit
Ultimate Tensile Strength	ASTM E8	1050	MPa
0.2% Yield Strength	ASTM E8	800	MPa
Elongation at Break	ASTM E8	5%	-
Tensile Modulus	ASTM E8	140	GPa
Hardness	ASTM E18	30	HRC
Relative Density	ASTM B923	96%	-

## Data Availability

Data will be provided upon request.

## Glossary

AB	As-built
AC	Air cooling
ADAM	Atomic diffusion additive manufacturing
AM	Additive manufacturing
BPE	Bound powder extrusion
EDM	Electrical discharge machining
HCF	High-cycle fatigue
HT	Heat-treated
LCF	Low-cycle fatigue
L-PBF	Laser-powder bed fusion
PH	Precipitation hardening
SEM	Scanning electron microscopy
SLM	Selective laser melting
SS	Stainless steel
